# Prevalence of coliform bacterial contamination in cat drinking water in households in Thailand

**DOI:** 10.14202/vetworld.2021.721-726

**Published:** 2021-03-22

**Authors:** Suttiporn Srikullabutr, Panpicha Sattasathuchana, Anusak Kerdsin, Naris Thengchaisri

**Affiliations:** 1 Department of Companion Animal Clinical Sciences, Faculty of Veterinary Medicine, Kasetsart University, Bangkok, Thailand; 2 Department of Community Health, Faculty of Public Health, Kasetsart University, Chalermphrakiat Sakon Nakhon Province Campus, Sakon Nakhon 47000, Thailand

**Keywords:** animals, cats, coliform, contamination, *Escherichia coli*, hygiene, prevalence, water

## Abstract

**Background and Aim:**

Bacterial contamination of drinking water is a leading cause of gastrointestinal infections. Cats may be at risk of water contamination from feces due to poor sanitation and hygiene. The objectives of the present study were to (1) evaluate the prevalence of coliform bacteria in cat drinking water and (2) identify possible risk factors leading to contamination.

**Materials and Methods:**

Fifty-five drinking water samples were collected from water containers used by cats (median age [range]: 5 years [8 months-15 years]) at their home. Using a sterile syringe, 50 mL water was collected directly from water containers. The water samples were stored in coliform enhancement media for 24 h and then submitted for bacterial culture.

**Results:**

The prevalence of fecal coliform contamination of cat drinking water was 67.27% (37/55; 95% confidence interval: 53.29-79.32%). There was no significant difference in the prevalence of coliform bacterial contamination of drinking water by age or gender of the cat or by water container type. However, bacterial contamination differed significantly between shorthaired cats and longhaired cats when comparing *Escherichia coli* (9/44 [20.45%] vs. 8/11 [72.72%], p<0.001) and *Enterobacter* spp. (16/44 [36.36%] vs. 9/11 [81.82%], p=0.007). For water that had been in a container longer than 12 h, there were significantly more contaminated tap water samples (16/19 [84.21%]) than contaminated processed water samples (9/17 [52.94%], p=0.047).

**Conclusion:**

Coliform contamination in cat drinking water is common and occurs more often in households with longhaired cats. Drinking water for cats should be changed every 12 h, especially for households using tap water.

## Introduction

Bacterial contamination of drinking water is a major cause of illness in humans, partly due to the increasing population size, which results in reduced water supply and quality for consumption [1]. The presence of large quantities of coliform bacteria in food and water indicates a lack of cleanliness and poor hygiene. Contamination of food, drinking water, and containers sby fecal coliform bacteria may cause food spoilage and dirty water, which can lead to illness. Coliforms are rod-shaped, Gram-negative, and nonsporulating bacteria that are often found in warm-blooded intestines. *Escherichia coli* is a major species of fecal coliform bacteria that play an important role in water contamination [2]. Most *E. coli* are not pathogenic microorganisms, but some strains of *E. coli* can cause gastroenteritis [3], and these strains are known as diarrheagenic *E. coli* (DEC) [4]. Consuming contaminated food or water containing this bacterial group can lead to food poisoning. *Enterobacteriaceae* species belonging to the genera *Enterobacter* spp., *Klebsiella* spp., and *Citrobacter* spp. are classified as coliform bacteria. The coliform bacterial count is used as an indicator of food and water [5-9] sanitation, because these bacteria are not generally found in clean water [10].

Clean water is essential for cats to maintain body moisture, organ structure (skin and internal organs), and body fluid levels [11]; regulate body temperature; and eliminate waste from their body through urine, sweat, and respiration [12]. Unsafe water sources, inadequate hygiene conditions, or exposure to feces-contaminated water may lead to gastroenteritis in cats. The prevalence of coliform bacteria in cat drinking water in Thailand has not been studied previously. In humans, DEC may cause a wide spectrum of symptoms, ranging from mild diarrhea to severe hemorrhagic colitis [13]. The main transmission methods of DEC include direct contact, consumption, and transfer of genes among bacteria. Transmission can be prevented by implementing strict sanitation procedures, practicing good hygiene, and filtering contaminated water before consumption.

The objective of this study was to evaluate the prevalence of coliform bacterial contamination of cat drinking water in households in Thailand. Factors influencing coliform contamination of cat drinking water (age, breed, sex, hair length, container type, water source, and time of sampling) were also determined.

## Materials and Methods

### Ethical approval and Informed consent

The protocol was approved by the Kasetsart University Institutional Animal Care and Use Committee (approval number #ACKU62-VET-030) and by the Ethical Review Board of the Office of National Research Council of Thailand (NRCT license U1-00500-2558). Written consent was obtained from all cat owners and the experiment complied with the Kasetsart University Institutional Animal Care and Use Standards.

### Study period and location

The study was conducted on 55 cats visiting Kasetsart University Veterinary Teaching Hospital, Bangkok, Thailand, from October to December 2019.

### Animals

Fifty-five cats (median age [range]: 5 years [8 months-15 years]; 38 males and 17 females; weight range: 1.8-7 kg) were enrolled in the study. There were 40 domestic shorthair cats, eight Persian cats, four British shorthair cats, and three Scottish fold cats. These cats were seen routinely at the cat clinic for follow-up visits to monitor chronic disease with various symptoms, including soft stool (fecal score of more than 4 of 7; 12/55 [21.82%]), and vomiting (5/55 [9.09%]), abdominal cramps (3/55 [5.45%]), fever (1/55 [1.82%]), anorexia (10/55 [18.18%]), and depression (6/55 [10.91%]).

### Water sample collection

This study was a randomized cross-sectional survey to identify the prevalence of water contamination by coliform bacteria among water samples collected from water containers in the cats’ homes. Cat drinking water was collected by the pet owner using a sterile 50-mL syringe. Details on water container type, water source, and frequency of water change were recorded.

### Coliform bacterial culture

Direct water culture was conducted by submitting 1 mL of water sample for bacterial culture using MacConkey agar. Indirect water culture was conducted by collecting 15 mL of water sample in coliform enhancement media containing tryptone, bile salt, X-GluC (a chemical compound with the molecular formula C_14_ H_13_ BrClNO_7_, 5-bromo-4-chloro-3-indolyl-beta-D-glucuronic acid, and cyclohexylammonium salt), and ortho-nitrophenyl-b-galactoside, and was stored for 24 h at 28±2°C. One milliliter of water in coliform enhancement media was transferred to MacConkey agar. Pink colonies were selected to identify the type of bacteria, including *E. coli*, *Enterobacter* spp., *Klebsiella* spp., and *Citrobacter* spp. using standard biochemical reaction tests, as previously described [14,15].

### Statistical analysis

Commercially available statistical software packages (JMP Pro 10, SAS Institute, Cary, North Carolina, USA; GraphPad Prism version 6.0, GraphPad Software, Inc., La Jolla, California, USA; STATA version 12, StataCorp, College Station, Texas, USA) were used for the statistical analyses. A Shapiro–Wilk W-test was used to assess the normality of the data. Student’s *t*-test was performed to compare age and body weight of the cats. The categorical variables, including age, breed, sex, hair length, container type, water source, and time of sampling were compared using Fisher’s exact test. The significance level was set at p<0.05.

## Results

**Figure-1:**
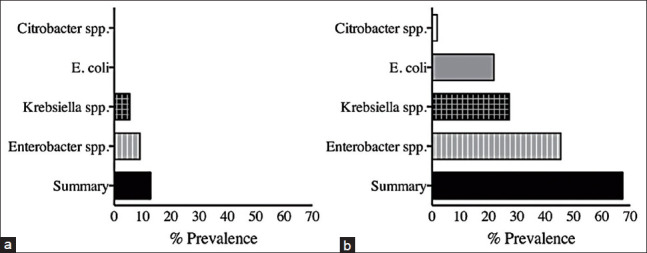
Prevalence coliform bacteria found in cat drinking water in the present study using (a) direct water culture and (b) indirect water culture.

Identification of bacterial contamination using a direct water culture resulted in the detection of a lower prevalence of water contamination (Figure-1a). The results of indirect water culture (Figure-1b) revealed a higher prevalence of bacterial contamination in cat drinking water. Out of the 55 water samples, 37 samples (67.27%; 95% confidence interval: 53.29-79.32%) were identified as having coliform contamination, including contamination with *E. coli* (12 [21.82%]), *Enterobacter* spp. (25 [45.45%]), *Klebsiella* spp. (15 (27.27%]), and *Citrobacter* spp. (1 [1.82%]) (Figure-1).

The distribution of bacterial contamination of drinking water by breed was as follows: Domestic shorthair (25/40 [62.50%]), Persian (8/8 [100.00%]), British shorthair (2/4 [50.00%]), and Scottish fold (2/3 [66.67%]) (Table-1). There was no significant difference in coliform bacterial contamination between the water of adult cats (<7 years; 29/42 [69.05%]) and senior cats (≥7 years old; 8/13 [61.54%], p=0.426). There was also no significant difference in coliform bacterial contamination in cat drinking water between male cats (25/38 [65.79%]) and female cats (12/17 [70.59%], p=0.490) (Table-1). The number of drinking water samples that were contaminated with coliform bacteria was not significantly different for shorthaired cats and longhaired cats when comparing *Klebsiella* spp. (10/44 [22.73%] vs. 5/11 [45.45%], p=0.130) and *Citrobacter* spp. (1/44 [2.27%] vs. 0/11 [0.00%], p=0.614) (Table-2). However, there was a significant difference in the bacterial contamination of water samples for shorthaired cats and longhaired cats when comparing *E. coli* (9/44 [20.45%] vs. 8/11 [72.72%], p<0.001) and *Enterobacter* spp. (16/44 [36.36%] vs. 9/11 [81.82%], p=0.007) (Table-2).

**Table-1:** Association of cat characteristics (age, breed, and sex) with coliform bacterial contamination in cat drinking water.

Category	No. positive	No. negative	% positive	p-value
Breed				
Domestic Shorthair	25	15	62.50	---
Persian	8	0	100.00	0.087
British Shorthair	2	2	50.00	0.504
Scottish Fold	2	1	66.67	0.692
Age group				
Adult (age<7 years old)	29	13	69.05	---
Mature (age≥7 years old)	8	5	61.54	0.426
Sex				
Male	25	13	65.79	---
Female	12	5	70.59	0.490

**Table-2:** Effects of coat length on coliform bacterial contamination of cat drinking water.

Type of coliform bacteria	Shorthaired cats (n=44)		Medium- and long-haired cats (n=11)		p-value
	
	No. positive	% Positive	No. positive	% Positive	
*Escherichia coli*	9	20.45	8	72.72	<0.001
*Enterobacter* spp.	16	36.36	9	81.82	0.007
*Klebsiella* spp.	10	22.73	5	45.45	0.130
*Citrobacter* spp.	1	2.27	0	0.00	0.614
Total	27	62.36	10	90.91	0.069

The number of plastic containers that contained water contaminated by coliform bacteria (24/35 [68.57%]) did not differ from the number of ceramic containers that contained contaminated water (7/11 [63.64%], p=0.761) or metal containers (6/9 [66.67], p=0.913) (Table-3). There was no significant difference in the number of tap water versus processed water samples contaminated with coliform bacteria when comparing *E. coli* (7/28 [25.00%] vs. 5/27 [18.52%], p=0.561), *Enterobacter* spp. (16/28 [57.14%] vs. 9/27 [33.33%], p=0.076), *Klebsiella* spp. (9/28 [32.14%] vs. 6/27 [22.22%], p=0.409), and *Citrobacter* spp. (1/28 [3.57%] vs. 0/27 [0.00%], p=0.322) (Table-4). The overall proportion of tap water samples contaminated with coliform bacteria (22/28 [78.57%]) did not differ significantly from that of contaminated processed water samples (15/27 [68.18%], p=0.383) (Table-4). There also was no significant difference in the contamination of tap water samples (6/9 [66.67]) versus the contamination of processed water samples (6/10 [60.00%], p=0.57) that had been stored in a container for up to 12 h (Table-5). However, there was a significant difference in the bacterial contamination of tap water samples (16/19 [84.21%]) and processed water samples (9/17 [52.94%], p=0.047) that had been stored in the container for longer than 12 h (Table-5).

**Table-3:** Proportion of cat drinking water samples positive for coliform bacteria, by container type.

Type of bacteria	Plastic container (n=35)		Ceramic container (n=11)		Metal container (n=9)		p-value
		
	No. positive	% Positive	No. positive	% Positive	No. positive	% Positive	
*Escherichia coli*	9	25.71	2	18.18	1	11.11	0.803
*Enterobacter* spp.	13	37.14	6	54.55	6	66.67	0.244
*Klebsiella* spp.	8	22.86	4	36.36	3	33.33	0.615
*Citrobacter* spp.	0	0.00	0	0.00	1	11.11	0.164
Total	24	68.57	7	63.64	6	66.67	1.000

**Table-4:** Proportion of cat drinking water samples positive for coliform bacteria, by water source.

Type of bacteria	Tap water (n=28)		Processed water (n=27)		p-value
	
	No. positive	% Positive	No. positive	% Positive	
*Escherichia coli*	7	25.00	5	18.52	0.561
*Enterobacter* spp.	16	57.14	9	33.33	0.076
*Klebsiella* spp.	9	32.14	6	22.22	0.409
*Citrobacter* spp.	1	3.57	0	0.00	0.322
Total	22	78.57	15	55.56	0.089

**Table-5:** Proportion of cat drinking water samples positive for coliform bacteria, by sampling time and water source.

Time of sampling (h)	Water source	Coliform bacterial contamination			p-value

		Number of positive	Number of negative	% positive	
<12 h	Tap water	6	3	66.67	---
	Processed water	6	4	60.00	0.57
>12 h	Tap water	16	3	84.21	---
	Processed water	9	8	52.94	0.047
Total	Tap water	22	6	78.57	---
	Processed water	15	12	55.56	0.089

## Discussion

The present study found that the prevalence of coliform bacteria contaminating cat water containers was between 53.29% and 79.32%. Water samples from households with a longhaired cat were associated with a significantly higher prevalence of coliform bacterial contamination, which was likely due to the fact that longhaired cats become more easily soiled by dirt and feces from cat litter. This study revealed evidence that processed water is less likely to be contaminated by bacteria (*E. coli* and *Enterobacter* spp.) compared with tap water. Tap water that had been left in the water container for more than 12 h had higher bacterial contamination compared with processed water.

*E. coli* is a normal flora in the intestines of humans and animals [16,17] and may serve as an indicator of food or water contamination by microbes from the digestive tract. *E. coli* can be used as an indicator of contamination events based on evidence that the bacteria cannot survive for long outside of the host nor create a biofilm habitat for regeneration [18]. The incubation period of *E. coli* is usually between 12 h and 8 days, and the duration of illness is usually between 1 day and 3 weeks. Detection of coliform bacteria in drinking water indicates recent contamination (within 1-2 weeks). Coliform-contaminated water should not be consumed, because there may be other pathogenic organisms present [19].

Bacterial contamination in food and water can result from direct or indirect contact with fecal materials. It is possible that contamination may have been caused by the cats. In the present study, *E. coli*, *Klebsiella* spp., *Enterobacter* spp., and *Citrobacter* spp. were identified in cat drinking water, which further confirmed the possibility that the drinking water was contaminated with fecal materials. Coliform bacterial contamination was found more often in the drinking water of longhaired cats. Thicker coats may trap more dirt and feces from cat litter. As such, insufficient grooming may lead to coat contamination and subsequent water contamination. Fecal contamination of water may be caused by other behaviors, such as when cats stir their water while drinking or otherwise dip their feet in the water bowl.

Several factors can influence bacterial contamination of drinking water, including pipe breaks, low-pressure conditions, and low-chlorine concentrations, which result in the intrusion of pathogenic microorganisms [20,21]. The results of bacterial cultures were also influenced by direct and indirect water samples. In the present study, the prevalence of water contamination in cat drinking water was based on the result of the indirect bacterial culture, since the direct bacterial culture of the water sample yielded significantly lower detection. The World Health Organization recommends that chlorine be present in sterile drinking water at a concentration of 0.2-1 mg/L. In the present study, we found that tap water that was left in the water container for more than 12 h was more likely to be contaminated than water that had been left in the container for less than 12 h. It is possible that the effectiveness of chlorine may be reduced when water stays in the container for more than 12 h. Therefore, it is advisable for cat owners to clean water containers and change the drinking water twice daily to prevent bacterial infections in cats.

In humans, lack of access to clean water sources has been linked to up to 4% of deaths worldwide [22]. Water contamination is a major cause of gastrointestinal diseases in humans. From 1990 to 2004, 86 pathogens were identified as contaminants in the public drinking water of the European Union [23]. In the United States, 780 types of infections were associated with drinking water from 1971 to 2006 [24], and every year, more than 20 million people are infected with waterborne diseases [25]. The occurrence of waterborne diseases is caused by many factors, such as an inefficient water system [26-28], inadequate disinfection systems [1,29], cracks in the pipes transporting the water source that link waste and drinking water [30], or decreases in the pressure of the water distribution system [20,21,31]. Consuming contaminated water can affect many people and pets in a short time [1,32]. Bacterial contamination in cat drinking water found in the present study may suggest the possibility of zoonotic transmission of waterborne diseases from cats to humans. Animal waste products are sources of waterborne pathogens [10]. Pathogens use wastes products as a means of transport from animal reservoirs to water environments. Humans may accidentally consume food or drinks that are contaminated with coliform bacteria [1] or other pathogens [27,28] after changing the cat drinking water or touching cat litter. It is important to wash hand with soap after changing cat water or cat litter. Hand washing helps prevent bacterial contamination and other zoonotic transmissions. More studies are needed to evaluate the influence of coliform bacterial contamination of cats’ drinking water on their general health status.

## Conclusion

The present study demonstrated that coliform bacterial contamination was more commonly found in the water containers of longhaired cats. The results of the present study suggest that processed water may be preferable to use as drinking water for cats, because it is less susceptible to water contamination compared with tap water. Owners should clean water containers and change the drinking water for cats twice daily, especially if they use tap water and/or have a longhaired cat.

## Authors’ Contributions

SS: Designed study, conducted literature review, performed study, interpreted data, and drafted manuscript. PS: Designed study and reviewed manuscript. AK: Performed study and reviewed manuscript. NT: Designed study, interpreted data, and reviewed manuscript. All authors read and approved the final manuscript.
